# Vitamin D and Temporomandibular Disorders: What Do We Know So Far?

**DOI:** 10.3390/nu13041286

**Published:** 2021-04-14

**Authors:** Andreea Kui, Smaranda Buduru, Anca Labunet, Silvia Balhuc, Marius Negucioiu

**Affiliations:** 1 Prosthodontics Department, Iuliu Hatieganu University of Medicine and Pharmacy, 400006 Cluj Napoca, Romania; andreeakui@gmail.com (A.K.); smarandabudurudana@gmail.com (S.B.); syylvya@yahoo.com (S.B.); negucioiu.marius@gmail.com (M.N.); 2 Dental Materials Department, Iuliu Hatieganu University of Medicine and Pharmacy, 400006 Cluj Napoca, Romania

**Keywords:** vitamin D, 25-hidroxyvitamin D, temporomandibular disorders, osteoarthritis

## Abstract

Background and aims. Vitamin D is synthesized in the skin with the aid of ultraviolet-B radiation, playing a variety of roles in the body. Temporomandibular disorders (TMDs) are a group of pathological conditions involving the temporomandibular joints as well as the masticatory muscles and othersurrounding tissues. In the present narrative review, we investigated the potential role of vitamin D in the etiology of temporomandibular disorders in order todetermine whether the current knowledge supports 25-hidroxyvitamin D (25-OHD) supplementation in temporomandibular disorders associated with insufficient or deficient levels of vitamin D. Methods. A literature research was performed in PubMed, Scopus, Science Direct, and Google Scholar databases, and a total of 10 articles were included for analysis. Results.Among the observational studies published to date, investigating the role for vitamin D in the etiology of TMDs, six of them suggest that there is a connection between the two aspects. In this context, patients suffering from TMD, with deficient levels of vitamin D (<30 ng/mL), are most likely to benefit from supplementation, whereas individuals with vitamin D level >50ng/mL probably have little benefit from supplementation.Conclusion.Vitamin D might be a safe, simple, and potentially beneficial way to prevent TMDs or to reduce pain; however, more randomized and placebo-controlled trials are required before any firm conclusions can be drawn.

## 1. Introduction

25-hidroxyvitamin D (25-OHD), also known as vitamin D, is a fat-soluble vitamin, synthesized in the skin (from a precursor), and obtained from dietary sources (e.g., oily fish, dietary supplements, or vitamin D-fortified foods). Among the causes of vitamin D deficiency, insufficient UVB exposure or decreased bioavailability are often mentioned, along with some medication, such as glucocorticoids, antiretroviral drugs, or anticonvulsants [1].

As the majority of human body tissues express vitamin D receptors [2], it is known that the lack of 25-OHD is implicated in a range of pathological conditions, including musculoskeletal disorders; metabolic, autoimmune, respiratory, and cardiovascular diseases; malignancies; psychiatric conditions; and chronic pain [1].

Temporomandibular disorders (TMDs) represent a group of pathological conditions involving the temporomandibular joints, the masticatory muscles, as well as the surrounding tissues and bone structures, or any combination of the structures mentioned above [3,4]. In 2014, an updated classification structure for TMDs was published by the International Research Diagnostic Criteria for Temporomandibular Dysfunction Consortium Network. As a result, four main categories of conditions were included, such as (a) temporomandibular joint disorders (intra-articular disorders), (b) mastication muscle disorders (extra-articular disorders), (c) headache (attributed to TMD), and (d) disorders of the associated structures [5] (Table 1).

Multiple factors are involved in the etiology of TMDs, including local and systemic conditions; additionally, biological, environmental, emotional, and cognitive factors are known to be triggers for this pathology [6]. Among the systemic diseases, rheumatoid arthritis, inflammatory conditions, ankylosing spondylitis, and immune diseases such as lupus are known to be implicated in TMDs [7].

The prevalence of TMDs among the general population is considered to be 5%; there are studies suggesting that approximately 5%–60% of the population experience at least one of the signs of TMDs [3,6]. Ryan et al. found in their research that the prevalence of TMDs peaks among people aged 25–45, and it seems that women are more affected than men. Among adolescents aged 14–18 years, TMDs have a prevalence of about 30% [7,8,9,10].

Over the last two decades, and especially during the last year [11,12,13,14], scientific interest in vitamin D has increased greatly. As the etiology and pathogenesis of TMDs arenot completely understood, the therapeutic options are not always successful. Therefore, understanding the etiology of temporomandibular joint disorders is important, as well as identifying and eliminating the potential pathogenic factors. The aims of this review areto examine the evidence to date regarding vitamin D and temporomandibular disorders and to investigate whether the current knowledge supports 25-OHD supplementation in temporomandibular disorders associated with insufficient or deficient levels of vitamin D.

## 2. Method for Literature Search

**Figure 1:**
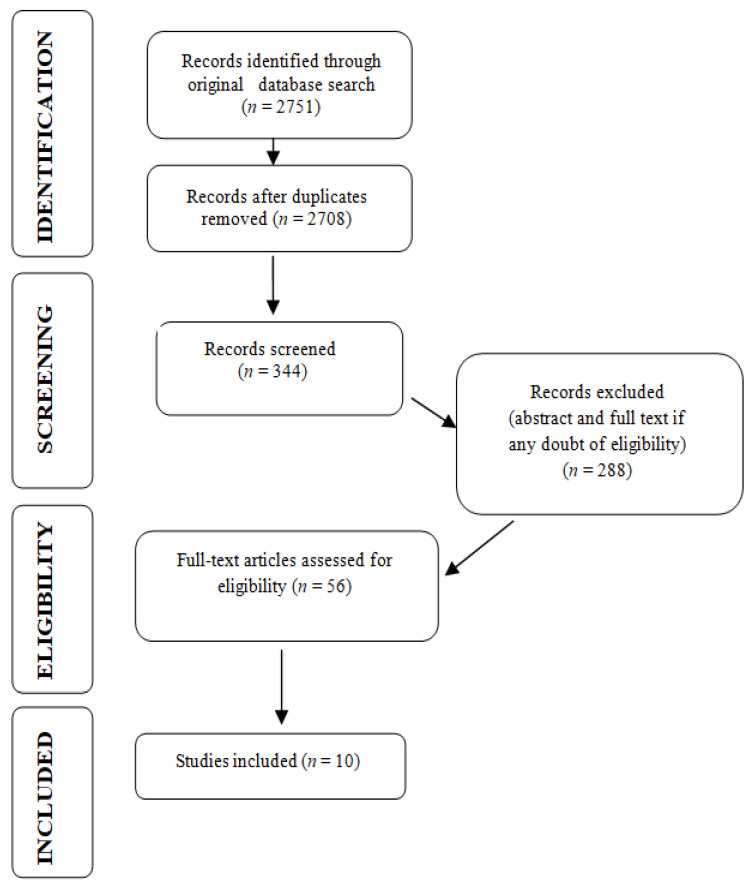
PRISMA flow diagram for research stages.

Serial literature searches for published studies of interest were performed between December 2020 and January 2021. PubMed, Scopus, Science Direct, and Google Scholar databases were searched using a combination of the following Medical Subject Headings (MeSH) terms:“vitamin D”, “temporomandibular disorders”, “masticatory muscles disorders”, “osteoarthritis”, and “bruxism”. Articles published between 2010 and 2021 were included. For this research, we considered the following to be eligible (all published in English language): clinical trials, randomized controlled trials, reviews, and systematic reviews.Exclusion criteria were non-human research, studies whereby outcomes of interest were not investigated, or articles written in languages other than English. A total of 2751 of records was identified, 43 duplicates were removed, 344 articles were screened, and 56 articles were assessed for eligibility. Studies investigating osteoarthritis localized on joints other than temporomandibular joints (TMJ) were not included in the narrative, and 10 studies were included in this review (Figure 1).

## 3. Current Status of Knowledge

### 3.1. Definitions

The implications of vitamin D in temporomanidbular disorders can be evaluated after careful consideration and understanding of different types of disorders, physiology and genetic variances involved in this interaction.

#### 3.1.1. Temporomandibular Disorders

Disk derangement disorders, osteoarthritis, autoimmune disorders, and myofascial pain disorder are some of the more common syndromes associated with temporomandibular disorders (Table 1). While musculoskeletal conditions are responsible for at least 50% of TMDs, osteoarthritis and disk displacement are the most frequent intra-articular disorders of temporomandibular joints (TMJs) [3,15,16].

Since pain is often the main symptom that determines the patients to seek medical aid, TMD diagnosis requires a thorough medical history and physical examination. This is because other conditions that mimic TMDs often challenge the clinician in establishing the differential diagnosis. Pathologic conditions such as dental lesions, oral abscess, or oral lesions, and conditions resulting from muscle overuse (e.g., bruxism, clenching, muscle spasm, and excessive chewing), trauma, maxillary sinusitis, salivary gland disorders, neuralgia, etc. are also associated with orofacial pain [3,16]. Therefore, in establishing a TMD diagnosis, other signs and symptoms should be emphasized—the limitation of mandibular movements and/or joint sounds. All the clinical findings associated with imaging studies (Panoramic X-rays, TMJ X-rays, CT scans, and MRI) and other complementary explorations (blood analysis and biochemistry of synovial fluid) lead to a specific diagnosis regarding the type of temporomandibular disorder [17].

Osteoarthritis represents a destructive process affecting temporomandibular joints [18]; however, it is considered that, at least in its initial phases, it has a non-inflammatory origin [19].The pathological mechanism behind this intra-articular TMD is defined by an articular cartilage degradation and abrasion, as well as a local thickening and remodeling of the underlying bone. Secondary inflammatory modifications are sometimes superimposed on top of these changes [19]. Among the clinical signs in the case of osteoarthritis, pain and reduced mobility of the jaw are the most frequent, regardless of the specific joint noises during mouth opening (crepitus) [15].

As the etiology of TMDs is multifactorial, their management protocol often involves a multidisciplinary approach. The initial aim of therapy should be resolving pain and dysfunction, while invasive treatments such as joint surgery should be reserved for non-responsive cases [3,20,21]. Among the conservative therapies, patient education and self-care, physical therapy, medication (non-inflammatory drugs and muscle relaxants), and the use of occlusal splints and occlusal adjustments are often indicated [3,14].

#### 3.1.2. Physiology and Actions of Vitamin D

Vitamin D is synthesized in the skin with the aid of ultraviolet-B radiation and converted to the active form 1,25-dihydroxyvitamin D in two steps, which binds to the vitamin D receptor (VDR) [22]. A large number of genes are regulated by the activated VDR complex. Compared with1,25-dihydroxyvitamin D (which has a half-life of four hours), the pro-form 25-hydroxyvitamin D has a half-life of about three weeks, and it is also more stable than the former. As a result, 25-OHD is used to determine the vitamin D status [23,24].

Whether synthesized endogenously in the skin after sun exposure or ingested in the diet, vitamin D (25-OHD) plays a role in a wide range of processes in the body.

Vitamin D is required for a healthy musculoskeletal system, as it plays an important role in calcium and phosphorus metabolism—not only by increasing their intestinal absorption and regulating the parathyroid hormone (PTH) production, but also by inducing the pre-osteoclasts to become mature osteoclasts [25,26,27].

Moreover, vitamin D is also known to regulate ~3% of the human genome through vitamin D receptor (VDR), which forms a heterodimer with the retinoid X receptor (RXR) [22,28]. Several types of tissue in the human body (such as pancreatic islets, prostate, macrophages, malignant, and immune cells) are capable of producing local 1,25-dihydroxyvitamin D as a result of possessing 1-α-hydroxylase [29,30]. This aspect suggests the role of vitamin D in preventing cancers, in affecting endocrine systems and insulin release, as well as in other metabolic processes.

Controversies still exist regarding the optimal serum levels of 25-OHD; however, over the last decade, an overall agreement was reached that circulating 25-OHD values below 30ng/mL are an indication of low vitamin D status [22]. Ideally, in deficient cases, vitamin D supplementation should be made according to the vitamin D serum levels (Table 2).

#### 3.1.3. Vitamin D and Genetic Variances

A large number of genes is regulated by the activated VDR complex, and since the *VDR gene* polymorphisms have been investigated, several associations have been evaluated with a wide variety of diseases, including musculoskeletal conditions, osteoarthritis, diabetes, malignant tumors, cardiovascular diseases, viral infections, tuberculosis, oral diseases, urinary stone, and autoimmune diseases [31,32].

It is known that the *VDR gene* is situated on chromosome 12 (*12q12-q14*). It has numerous single nucleotide polymorphisms (SNPs) (*gene* polymorphisms sites) and expresses a ligand-activated transcription factor [33,34]. Some of these SNPs play critical roles in the modification of 1,25-dihydroxyvitamin D uptake [17] and can promote vitamin D function and metabolism [33]. As both the non-coding and coding regions of VDR’s sequence have several biallelic polymorphic sites, they can influence the rate gene transcription, the quality of the resulting protein, or the stability of mRNA. Therefore, it is considered that predisposition or disease severity might be linked to these sites [35].

The *VDR gene* contains over 100 restriction polymorphic sites, the most well-known of which are *ApaI* (*rs7975232*), *TaqI* (*rs731236*), *BsmI* (*rs1544410*), and *FokI* (*rs1544410*) [36]. Several studies have investigated the links between the *VDR* polymorphism and cartilaginous tissue diseases (such as osteoarthritis of knee, hip, or inter-vertebral disks, lumbar disks, etc.), and a large majority of them revealed an association between them.

In 2018, Yilmaz et al. investigated whether *VDR* polymorphism also presents susceptibility to internal derangements in temporomandibular disorders. Performing a clinical study on 119 patients, the authors could not reveal any association between *VDR TaqI* and *ApaI gene* polymorphisms and intra-articular temporomandibular disorders [32].

In 2020, Yildiz et al. published a research investigating the relationship between vitamin D and a *Bsml* variant with TMD, specifically, disk displacements with reduction (DDR). The case–control study included 104 Turkish patients diagnosed with DDR and 102 healthy Turkish individuals, investigating a *Bsml* variant of VDR. The authors concluded that the *Bsml* variant of the *VDR gene*, as well as the low levels of vitamin D, plays a role in the etiology and pathogenesis of disk displacements with reduction [37].

### 3.2. Vitamin D and Temporomandibular Disorders

It has been shown that vitamin D plays a significant role in the musculoskeletal and cardiovascular systems, as well as in the control of calcium and phosphate metabolism and the maintenance of sufficient blood levels of these minerals [38,39]; it seems that this nutrient is also related to cartilage regeneration in people with osteoarthritis (OA), although the mechanism is still unclear [40]. In the context of investigating a possible association between vitamin D and temporomandibular disorders, 10 articles were included in this literature review (Table 3).

In 2019, Madani et al. published a study that aimed to identify whether among the multiple factors involved in calcium metabolism, vitamin D is associated with temporomandibular disorders [41]. A total of 51 patients with different types of TMDs were included in their study, who were compared with 29 healthy subjects. Blood sample analysis revealed no significant association between the calcium-metabolism-related factors (such as serum phosphate, alkaline phosphate, parathyroid hormone (PTH), and vitamin D concentrations) and temporomandibular disorders.

Another study, published by Yilmaz et al. in 2019 [42], suggests a prevalence of TMDs (especially the muscular disorders) among chronic kidney disease (CKD) patients (~41.5%); however, due to the limitations of their study, the authors could not establish a cause and effect relationship between TMD and hemodialysis patients. These results are significant for future studies on vitamin D, which is considered to be deficient in CKD patients [22,43] and may play a role in the evolution of temporomandibular disorders on hemodialysis patients by inducing inflammatory cytokine production.

An exploratory study, published in 2017 by Khanna et al., investigated the influence of vitamin D on the temporomandibular joints and the daily activities of a population in Mumbai. The research revealed an association between TMJ pain and discomfort, affecting also the daily activities of the subjects’ daily activities [44]. Although those results confirm similar findings published earlier by Shen et al. and Jagur et al. [45,46], future studies (which might investigate the influence of vitamin D on TMJ health post-supplementation) should be carried out in order to establish a link between the two aspects.

Those results were also confirmed by Yilmaz and Ersoz in 2018 [47], whose research investigated the biochemical changes in patients diagnosed with TMDs. The serum levels of vitamin D, parathyroid hormone, calcitonin, calcium, phosphorus, and magnesium were investigated in a group of TMDs patients, compared with a control group. A total of 100 subjects were included in this prospective study, with a total of 50 patients diagnosed with TMDs and intra-articular pathologies. The results revealed no significant difference inthe serum levels of vitamin D, calcium, phosphorus, and magnesium between the two groups; however, the levels of parathyroid hormone in response to vitamin D deficiency weresignificantly higherin the study groups (TMDs subjects) compared to the control group. The authors concluded that, in patients with TMDs, the vitamin D serum level should be investigated, and a correction should be performed in case of a deficiency.

Different results were obtained by Staniszewski et al. [48]. In their research, published in 2019, a serum analysis was performed on a group of patients with temporomandibular disorders compared with a group of healthy subjects. They investigated several common variables (such as blood cell count, TSH, FT4, PTH, creatinine, sodium, potassium, calcium, albumin, and vitamin D.). The authors concluded that there was no clear evidence of systemic disease or malnutrition in the TMD group. Although the results might implythat serum analysis should not be used as a biomarker in the case of temporomandibular disorders, the investigation also revealed the fact that the administration of high doses of vitamin supplements in a group of TMD patients contributed to elevated serum levels in some variables.

As inflammatory arthritis (especially rheumatoid arthritis) is also known to have an affinity to temporomandibular joints, Ahmed H. S. published a study in 2017, investigating the serum levels of some variables (including vitamin D, calcium, and interleukin-1) in 45 TMD patients suffering from rheumatoid arthritis. Comparing the results with a group of healthy individuals, the research revealed that the vitamin D levels were significantly lower in TMD patients. The results also showed a significant increase in serum total alkaline phosphatase as bone marker and interleukin-1 in TMD patients. Their research also revealed that vitamin D might exert anti-inflammatory effects by diminishing the release of pro-inflammatory cytokines, as well as by inhibiting T-cell response [49].

Several oral habits and parafunctions are considered to be involved in the etiology of temporomandibular disorders. Bruxism is a rhythmic masticatory muscle activity, characterized by teeth grinding and clenching, which can occur during sleep or while awake [50].In 2021, Alkhatatbeh et al. published a case–control study, investigating whether there is an association among the low intake of dietary calcium, low levels of vitamin D, headache, and psychological symptoms (such as anxiety and depression) and sleep bruxism. A total of 100 subjects were enrolled in this observational study, and the results showed that, compared with the control groups, the subjects with sleep bruxism had higher anxiety and depression rates, and their vitamin D levels were significantly lower [51].

## 4. Vitamin D and Pain Management

The International Association for the Study of Pain describes pain as “an unpleasant sensory and emotional experience associated with actual or potential tissue damage, or describes in terms of such damage” [49,52]. There is a multitude of published studies investigating the relationship between vitamin D and different types of pain; especially for patients suffering from osteoarthritis, including TMD osteoarthritis, pain is a common symptom. In the Cochrane review, published in 2015, Straube et al. concluded that, based on the current evidence, for chronic painful conditions in adults, vitamin D supplementation might not be beneficial in terms of pain relief.The authors also mention that, in the clinical context, deficient levels of vitamin D should be corrected by treatment even thougha significant reduction of pain intensity (at least 50% reduction) should not be expected [1].

Headache is also an important symptom for patients suffering from temporomandibular disorders. According to the third edition of the International Classification of Headache Disorders (ICHD-3), the headache attributed to temporomandibular disorders is diagnosed in association with painful pathological conditions of TMJs and mastication muscles. Usually, this type of headache is most prominent in the temporalis, masseter muscle, and preauricular area. When the temporomandibular disorder is uncertain, tension type headache is a differential diagnosis (caused by the tenderness of pericranial muscles) [53].

In a literature review published in 2020, Nowaczewska et al. investigated the role of vitamin D in primary headaches [54], emphasizing the strong correlation between vitamin D deficiency and chronic musculoskeletal pain; patients with tension type headache seem to have low levels of vitamin D, and there is also evidence that patients with chronic headache have lower melatonin levels, with decreased nocturnal serum melatonin levels during cluster periods [55]. As vitamin D acts like a neuroprotective agent (it is involved in regulating the production of several neurotrophic factors, activates different enzymes in brain, and influences the neurotransmitters), Nowaczewska et al. concluded that there is important evidence in establishing a connection between the serum levels of 25-OHD and migraines but also with other types of headaches through different mechanisms.

Similar conclusions have also been drawn by Wu et al. in a systematic review published in 2018, emphasizing an association between the low serum levels of vitamin D and, in the case of arthritis, muscle pain and chronic widespread pain [56].

Several studies published to date have investigated the relationship between vitamin D serum levels and other types of pain. The results of a clinical study performed on 175 patients with knee osteoarthritis, published in 2017 by Manoy et al, revealed that supplementation with 40,000 IU of vitamin D2 (ergocalciferol) for 6 months improved grip strength and physical performance, although it did not improve knee extensions. The results also showed that vitamin D supplementation reduced oxidative protein damage, reduced pain, and improved the quality of life. Nevertheless, the authors conclude that it remains unclear whether vitamin D supplementation influences musculoskeletal pain or not [57].

In 2019, Park published a literature narrative review, which focused on the potential link between vitamin D and osteoarthritis (OA) by analyzing different types of data, including experimental or in vitro studies. Based on the research, Park concluded that vitamin D may have a preventative effect on joint pain, although clinical observations showed that it has little effect on cartilage volume loss or radiologic OA initiation. The effect of vitamin D may also differ according to the disease state [58].

In 2020, Jin et al. published a systematic review investigating the effects of vitamin D supplementation on pain and physical function in patients with knee osteoarthritis. Based on their analysis, the authors conclude that despite the fact vitamin D supplementation may improve pain and function in patients with knee osteoarthritis, the evidence from randomized clinical trials (RCTs) was controversial, because not all the RCTs measured the potential moderators of interest, or, not all of them were measured in the same way. Nevertheless, the results published by Jin et al. can provide critical evidence to help clinicians make subgroup-specific treatment decisions in order to increase therapeutic efficacy [59].

**Figure 2:**
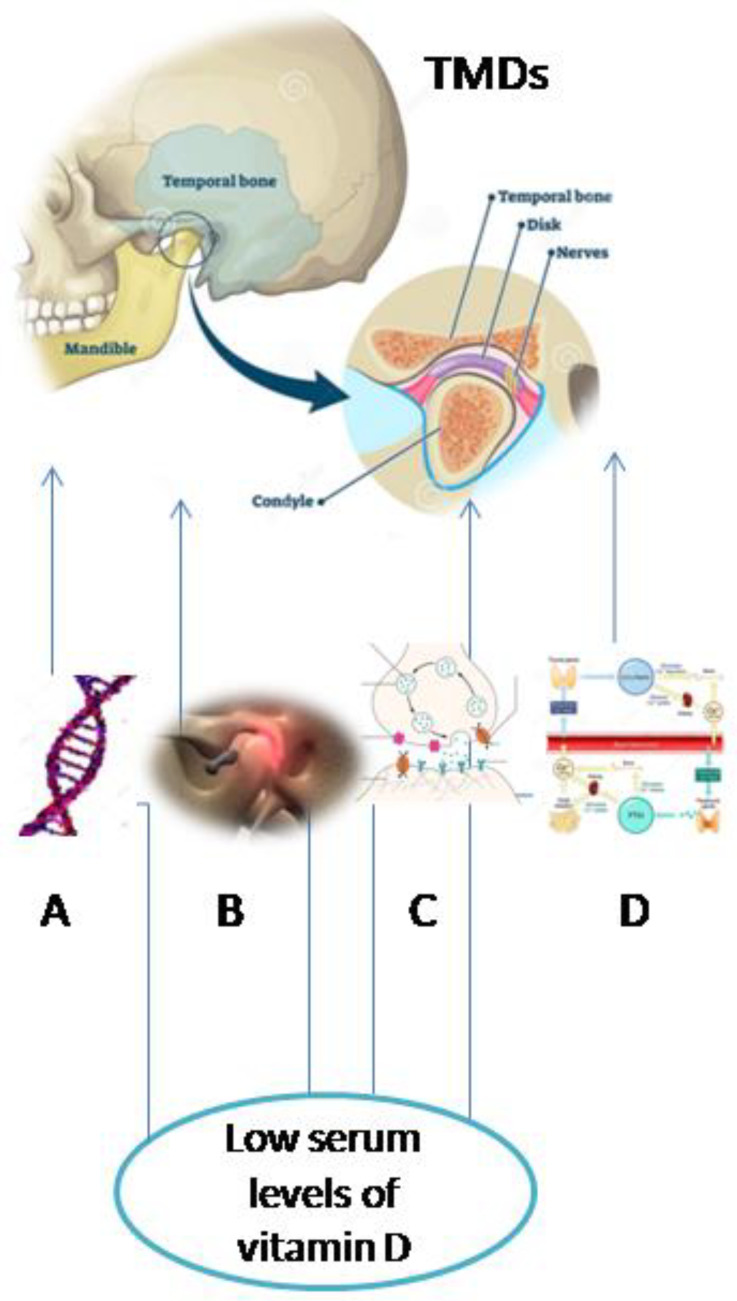
Possible pathways of interconnection between temporomandibular disorders and low levels of vitamin D (A—*VDR gene* polymorphism, B—inflammatory processes, C—neuroprotective function, and D—calcium and parathyroid hormone (PTH) metabolism).

Overall, this research revealed the different directions in which temporomandibular disorders might be associated with low levels of vitamin D. This is because vitamin D influences the calcium and PTH metabolism, has anti-inflammatory functions by diminishing the release of pro-inflammatory cytokines as well as by inhibiting T-cell response, and acts like a neuroprotective agent. Additionally, the *Bsml* variant of the *VDR gene* has been associated with temporomandibular disorders, specifically, disk displacements with reduction (Figure 2).

## 5. Conclusions

There are few observational studies showing an association between vitamin D deficiency and intra-articular temporomandibular disorders. There are several randomized controlled trials revealing a positive effect of vitamin D supplementation on pain management and on the quality of life for patients suffering from osteoarthritis. To date, there are no studies investigating the possible positive effects of vitamin D on pain management or on the prevention of temporomandibular disorders.

As clinical studies published to date are limited in their duration to study the role of vitamin D on TMDs prevention, vitamin D deficiency might be investigated and corrected. Although the evidence is weak for making general recommendations for vitamin D deficiency in cases of temporomandibular disorders, patients with vitamin D level <30nmol/L could benefit from supplementation. Therefore, vitamin D supplements might be indicated as an independent therapy for deficient patients with temporomandibular disorders.

Further well-designed randomized controlled trials are needed in order to confirm the relationship between vitamin D levels and the different types of temporomandibular disorders, or whether normal levels of vitamin D might prevent these disorders.

## Figures and Tables

**Table 1:** Classification of intra-articular and extra-articular temporomandibular disorders.

**INTRA-ARTICULAR** **DISORDERS**	**1. Joint Pain**	Arthralgia/Arthritis/Osteoarthritis		
	**2. Joint disorders**	Disk disorders/disk displacement	with reduction with intermittent locking without reduction, with limited locking without reduction, without limited locking	
		Other hypomobility disorders	Adhesions Adherence Ankylosis (fibrous/osseous)	
		Hypermobility disorders	Dislocations (subluxation/luxation)	
	**3. Joint diseases**	Systemic arthritis/condylysis/osteonecrosis		
		Degenerative joint disease/osteochondritis dissecans/ synovial chondromatosis/neoplasm		
	**4. Congenital disorders**	Aplasia/hypoplasia/hyperplasia		
**EXTRA-ARTICULAR** **DISORDERS**	**1. Muscle pain**	Myalgia/ tendonitis/ myositis/spasm		Local myalgia/myofascial pain/ myofascial pain with referral
	**2. Contracture**			
	**3. Hypertrophy**			
	**4. Neoplasm**			
	**5. Movement disorders**	Orofacial dyskinesia/oromandibular dystonia		
	**6. Masticatory muscle pain due to systemic pain disorder**	Fibromyalgia Widespread pain		

**Table 2:** Definition of vitamin D deficiencies and recommended supplementation with cholecalciferol.

Serum Vitamin D Levels	<5 ng/mL	5–15 ng/mL	16–30 ng/mL
**Definition**	Severe vitamin D deficiency	Mild vitamin D deficiency	Vitamin D insufficiency
**Dose of cholecalciferol**	8000 IU/day orally or enterally for 4 weeks, followed by 4000 IU/day	4000 IU/day orally or enterally for 12 weeks	2000 IU/day

**Table 3:** Overview of the articles investigating an association between different types of temporomandibular disorders and low level of vitamin D.

First Author and Year	Type of Study	Sample Size	Results
Yilmaz, A. D., 2018 [32]	Clinical Trial	119 subjects	No association between the *VDR TaqI* and *ApaI gene* polymorphisms and intra-articular TMDs.
Yildiz, S., 2020 [37]	Case–control Study	206 subjects	*Bsml* variant of *VDR gene*, as well as low levels of vitamin D, plays a role in the etiology and pathogenesis of intra-articular TMDs (disk displacement with reduction).
Madani, A., 2019 [41]	Case–control study	80 subjects	No significant association between vitamin D serum concentration levels and TMDs.
Yilmaz, F., 2020 [42]	Case–control study	146 subjects	High prevalence of TMDs among chronic hemodialysis patients.
Khanna, S., 2017 [44]	Case–control study	100 subjects	Vitamin D serum level had a significant impact on the TMJ pain and discomfort.
Jagur, O., 2011 [46]	Case–control study	95 subjects	TMJ radiographic changes and teeth loss seem to be related to the low level of bone mineral density and vitamin D serum levels.
Demir, C. Y., 2019 [47]	Clinical trial	100 subjects	Vitamin D status was similar between patients with TMDs and control group; increased parathyroid hormone levels in response to vitamin D deficiency were significantly higher in patients with TMDs.
Staniszewski, K., [48]	Controlled Cross-Sectional Study	120 subjects	Serum analyses should not be used as a biomarker of TMDs.
Ahmed, H. S. [49]	Clinical trial	45 subjects	Vitamin D levels were significantly lower in TMD patients with RA.
Alkhatatbeh, M. J., [51]	Case–control study	100 subjects	Vitamin D levels were significantly lower in subjects with sleep bruxism.

VDR—vitamin D receptor; TMDs—temporomandibular disorders; TMJ—temporomandibular joint; RA—rheumatoid arthritis.

## Data Availability

Not applicable.
